# Superficial Decay

**Published:** 1860-03

**Authors:** Geo. S. Fouk

**Affiliations:** New York


DENTAL REGISTER OF THE WEST.

VOL. XIII.] MARCH, 1860. [No. 9.

### Original Essays and Communications.

### SUPERFICIAL DECAY.

BY GEO. S. FOUKE.

THE term "superficial" is a dental technicality, the signification of which should be clearly comprehended. What is "superficial decay?" Is it synonomous with *incipient* decay? Not necessarily. Slight or shallow decay located on the surface of the enamel, externally, and not penetrating the substance of the enamel tissue, is properly *superficial* decay. Decay just beginning or commencing, either on the surface of the enamel, or at any appreciable point beneath the surface, is *incipient* decay.

A mere "roughness" marks the incipient stage of *superficial* decay, but if the disintegration advances materially into the substance of the enamel, or reaches the confines of the dentinal tissue, the affection should then be no longer recognized as superficial decay, but should be considered "deep-seated."

This distinction recognized, we should then distinguish the first stage of decay on the external surface, as *incipient* superficial decay, and the first stage of decay, not clearly *on* the surface, but obscurely located *interiorly*, we should denominate *incipient deep-seated decay.*

All decay, according to the generally received opinion, is from the surface, and beginning on the outside of the tooth. This opinion we do not design here to controvert. We would barely remark that the attack of decay, and the entire *res gestæ* (things in progress,) of its destructive march, are eminently *suggestive!* The enemy is both insidious and powerful! These are phenomena attendant upon the commencement and progress of dental decay, which, practically, are *just as good* as if caries actually begun its attack *interiorly* and not exteriorly on the surface of the enamel!

Decay may have its starting point in a very minute "congenital" defect *on* the enamel, but before the "weak point" is discovered the whole *internal* bone and considerable of the enamel in the *dentinal* regions are "irremediably" destroyed.

A *congenital* defect "in" the substance of the enamel, or even *in* the substance of the dentine, may not be after all a pathological impossibility! A defect may be so situated that whilst it reaches the surface, it may also extend simultaneously to the *interior* of the tooth. If *caries* commences its attack in this obscure defective portion of the enamel, it should not be regarded as a merely incipient superficial decay, it would be better to look upon the species of dental caries as *deep-seated,* and denominate it *incipient* deep-seated decay.

The proper treatment for *incipient superficial decay* is, unnqestionably, *filing.* Here the instrument is "invaluable!" Establish the certain existence of "superficial decay," in the only sense it should be recognized, and it would be safe to lay it down as "a rule," that teeth *should* be filed for "the removal" of such existing decay.

It is scarcely necessary to remind the dental practitioner that the treatment must be *timely!* In "few" cases does the decay remain long "superficial!" If it be taken, however, whilst it is *slight* or *shallow,* or clearly confined to the external surface, there would *not* be "comparatively few cases of superficial decay presented to our notice calling for the employment" of the file. Their number, me thinks, would be "legion."

In the treatment of superficial decay by filing, it is thought "age," "temperament," "habits of cleanliness," etc., should largely control or "govern" our action. *These* "considerations" may determine "the conditions *un*favorable," and, "if the teeth are not sufficiently decayed for filling," the course recommended is to "cleanse and burnish," and *wait!* We must *watch*, and when the proper time arrives for "filling," *wedge* "the teeth apart."

We have treated superficial decay on the approximal surfaces of *temporary* incisors at the age of *two*, *three* and *four* years, by carefully removing the affected parts with a *file* or *excavator*.

"Age" leaves a wide margin! So does "temperament," and also "habits in regard to cleanliness!"

"First class" teeth may be very properly regarded as the "fittest subjects for the file," in the removal of decay when it is superficial. But we recognize *so many classes* and *grades* of teeth, (nearly as many as there are grades and varieties in the human *features*,) that we can not help but believe that there are many teeth that are not *first class* that would be greatly benefited, if not permanently secured by employing "judiciously" upon them the file for removing incipient evils when existing in the form of superficial decay! It has been our lot to see "hundreds" aye, thousands of teeth that were saved by a "judicious" use of the file!

The treatment proper in *deep-seated* decay, in its *incipient* stage, must be determined by circumstances. *Filing* is seldom the course indicated. There are instances of rather extensive decay having been permanently arrested by *filing alone*. We remember one remarkable case.

In 1848 we were practicing in our native county (*Jefferson, Virginia.*) A young lady, aged about 20, had us to *examine* her teeth *prudentially*. Her teeth presented to the observer a fine appearance—no one would hardly "dream" a dentist had touched them! The incisors were slightly separated! We placed a glass in the lady's mouth, and we were never more surprised, dentally, than we were at the picture our eyes beheld! The appearance of the four upper incisors was then daguerreotyped on our mind never to be erased! All the approximal surfaces were *filed* to an almost incredible extent. Each tooth was filed diagonally till the planes of the filed surfaces came to within one *sixteenth* of an inch of each other, on the lingual or the palatine surface of the enamel. The *dentine* was clearly filed in the operation. The clear, firm, polished, healthy aspect of the filed surfaces, vied with the bright and beautiful enamel that covered her teeth! Our exclamation upon seeing the teeth was, "Why your teeth are filed!" Our fair visitor remarked, "Oh yes, Dr. K. filed my teeth eleven years ago!" It is now twelve years since we saw the lady, not having seen her since the occasion we are speaking of. If the lady is still living we feel confident she still has those remarkably filed incisors in her mouth.

This case impressed us deeply as to the *invaluableness* of the file in removing and arresting dental decay, but *we* have seldom ventured upon such a "bold" employment of the instrument! It has been a "model," however, that we have imitated in some cases!

In the case of *molars* and *bicuspids*, when decayed on their approximal surfaces, great caution is required in deciding whether to file or not, in their treatment. The certainty of the existence of purely *superficial* decay is generally very obscured, and the conformation of the teeth themselves is against filing. The "V" shape usually practiced is decidedly objectionable, and a *diagonal* cut requires too great a quantity of the solid enamel to be removed on the palatine sides.

We had a case last week (January 25th), of an evident attempt to save two *bicuspids*, by filing for what was no doubt supposed to be *superficial* decay, but which proved to be decay of a *deep-seated* kind. We give the case illustratively, in the hope it may be instructive :

Miss P. had the second bicuspids of the upper jaw treated for decay in the front approximal surfaces, by a dentist in a neighboring city. The time of treating the case was in August last. We had examined the teeth a few months previously. The teeth were filed and plugged in the month mentioned, but the plugs came out shortly after they were inserted. We were called upon to *re-fill* these teeth. We found that the file had been used very *elaborately*, a large "V" shaped space existing. It struck us an attempt had first been made to remove the decay simply by filing, but the cavity of decay proving to be too deep, plugging was then resorted to. But the worst kind of cavity, in consequence of the extensive use of the file, had now to be filled, and both plugs came out in a short time.

Had a different course been adopted in the treatment of these teeth, we feel no doubt but they could have been preserved. The teeth can be only saved now by extirpating the nerves, and proceeding as such treatment requires. The teeth are much "mutilated."

In further illustration of this subject, namely, the treatment of what may be supposed to be only superficial decay in *bicuspids* and *molars*, we will present a case that we treated *this day*:

Mr. N. R. brought to us this morning his daughter, aged 16 years, to have one tooth "filed." The teeth of this young lady are good—not far from *first class*.

"Something was wrong" with the lower first permanent molar. The "difficulty" was between it and its neighbor bicuspid. Too close "to thrive!" The parent thought "filing would do."

We *dissented*. Things looked pretty fair externally. But we suspected a "deeper-seated" *difficulty* than was supposed by the anxious parent to exist.

We chose to use the *drill.* Placing a flat drill about one sixteenth of an inch from the front approximal edge on the masticatory surface of the molar, we drilled through enamel apparently sound. A large cavity was soon laid open which we plugged. The other teeth all looked fine and seemed sound. We suggested that perhaps the corresponding molar on the other side of the jaw was similarly affected, though to a much less extent. The parent thought that if there was any thing wrong with that tooth it must be very slight decay, and he thought "filing would be all the tooth required." The deep-seated cavity found to exist in the one tooth, made us apprehensive as to the nature of the decay in the other if it really existed at all, and we determined to use the *drill* first. By the use of a sharp chisel we made a point for the drill in the enamel on the masticatory surface, as near to the approximal edge as was convenient to be done. The drill passed through solid enamel at least one *twelfth* of an inch, at which depth we found decay existing. We excavated and chisseled with very little difficulty to the depth of an eighth of an inch, or more, following the decay in the direction of the pulp cavity. The decayed portions removed were at first whitish and crumbly, but at the bottom of the cavity it assumed a bright, mahogany color and became tough and gristly. The adjoining bicuspid was unaffected; and the enamel on the approximal surface of the affected molar was very slightly decayed. We cut away the enamel, however, down to the gum or nearly so. A good sized cavity was formed which we filled with gold and dismissed the patient.

Now by "probing" we might have detected here a clear case of "superficial decay," which could have been removed with the file!

The "drill," however, laid open the obscure and extensive evil existing, and proper treatment was readily indicated, and the proper operation most effectually performed.

